# Association of high proprotein convertase subtilisin/kexin type 9 antibody level with poor prognosis in patients with diabetes: a prospective study

**DOI:** 10.1038/s41598-023-32644-y

**Published:** 2023-04-03

**Authors:** Hiroki Yamagata, Aiko Hayashi, Yoich Yoshida, Masaya Koshizaka, Shunichiro Onishi, Tomohiko Yoshida, Takaki Hiwasa, Minoru Takemoto

**Affiliations:** 1 Department of Diabetes, Metabolism and Endocrinology, School of Medicine, International University of Health and Welfare, 4-3, Kozunomori, Narita, Chiba 286-8686 Japan; 2 International University of Health and Welfare, Narita Hospital, Chiba, Japan; 3 Department of Endocrinology, Hematology, and Gerontology, Chiba University Graduate School of Medicine, Chiba, Japan; 4 Department of Neurological Surgery, Graduate School of Medicine, Chiba University, Chiba, Japan

**Keywords:** Biomarkers, Medical research

## Abstract

In addition to pathogenic autoantibodies, polyclonal autoantibodies with unknown physiological roles and pathogenicity are produced in the body. Moreover, serum antibodies against the proprotein convertase subtilisin/kexin type 9 (PCSK9) protein, which is integral to cholesterol metabolism, have also been observed. PCSK9 was also reported to be associated with insulin secretion and diabetes mellitus (DM). Therefore, we aimed to examine the clinical significance of PCSK9 antibodies (PCSK9-Abs) levels. We measured blood PCSK9-Abs and PCSK9 protein levels in 109 healthy donors (HDs) and 274 patients with DM (type 2 DM: 89.8%) using an amplified luminescence proximity homogeneous assay-linked immunosorbent assay. Subsequently, patients with DM were followed up (mean: 4.93 years, standard deviation: 2.77 years, maximum: 9.58 years, minimum: 0.07 years) to examine associations between antibody titers and mortality, myocardial infarction, stroke onset, and cancer. The primary endpoint of this study was to examine whether PCSK9-Abs can be a prognostic marker for overall mortality among the patients with diabetes. The secondary endpoint was to examine the relationship between PCSK9-Abs and clinical parameters. Although both PCSK9-Abs and PCSK9 protein levels were significantly higher in the DM group than in the HD group (p < 0.008), PCSK9-Abs and PCSK9 protein levels showed no correlation in either group. Mortality was significantly associated with higher PCSK9-Ab levels, but unrelated to PCSK9 protein levels. After investigating for potential confounding factors, higher PCSK9-Ab levels were still associated with increased mortality among the patients with DM. PCSK9-Abs may be a novel prognostic marker for overall mortality in patients with diabetes, and further studies are warranted to verify its usefulness.

## Introduction

In addition to autoantibodies related to autoimmune diseases, polyclonal autoantibodies of unknown physiological roles and pathogenicity are produced in the body. The roles of these autoantibodies are unclear; however, they may reflect the disease status of individuals. In fact, certain autoantibody types have been associated with arteriosclerotic diseases^1–3^ and malignancies^4,5^. Therefore, these autoantibodies can serve as novel biomarkers. We focused the proprotein convertase subtilisin/kexin type 9 (PCSK9) protein and PCSK9 antibodies (PCSK9-Abs). In 2003, *PCSK9* was the third most common causative gene for familial hypercholesterolemia^6^ and was found to promote degradation of the low-density lipoprotein (LDL) receptor^7,8^. Gain-of-function mutations in the *PCSK9* gene result in increased levels of blood PCSK9, degradation of LDL receptors, and increased levels of blood LDL. In contrast, loss-of-function mutations in the *PCSK9* gene result in stabilization of LDL receptors and decreased levels of blood LDL^9,10^. Regardless of the gene mutation, an increase in the levels of serum PCSK9 protein leads to an increase in the levels of LDL cholesterol (LDL-C), which may play a role in the development of arteriosclerotic diseases. However, the results of cross-sectional studies examining the association between blood PCSK9 protein levels and arteriosclerotic diseases are conflicting^11–14^.

Recently, it has also been reported that PCSK9 activity was positively correlated with insulin resistance in both patients with or without type 2 diabetes^15^. Indeed, increased levels of insulin stimulate PCSK9 production^16^. Further, PCSK9 knock out (KO) mice showed increased expression of the LDL-receptor (LDL-R) not only in the liver but also in the pancreas^17^. It has also been reported that increased expression of LDL-R in the pancreas of PCSK9 KO mice increased LDL-C influx to β cells, leading to impaired insulin secretion and eventually causing glucose dysmetabolism^17^. Even in clinical settings, PCSK9 protein levels are reportedly increased in type 2 diabetes patients^18–20^. These results indicate that PCSK9 is related not only with lipid but also glucose metabolism. The prevalence of diabetes mellitus (DM) is increasing worldwide^21^, and the patients with DM are at an increased risk of developing atherosclerotic cardiovascular disease and death^22^. Thus, it is imperative to develop a biomarker that can be used as a prognostic indicator for overall mortality, especially in DM.

Some large clinical trials have indicated that the effects of initial intensive treatment for hyperglycemia are maintained for several years and have beneficial effects on the cardiovascular disease, regardless of glycemic control in the later course of DM. This phenomenon is called glucose metabolic memory^23^.

Since it is known that the infectious memory carried by antibodies is largely responsible for protecting the body from reinfection, we hypothesized that the PCSK9-Ab acts as a metabolic memory and might be used as a prognostic marker for overall survival. Therefore, we aimed to determine PCSK9-Ab titers in healthy donors (HDs) and patients with DM and evaluate their clinical significance.

## Methods

This was a prospective study conducted in one facility in Japan.

Blood samples were obtained from 109 HDs, at the Port Square Kashiwado Clinic, and from a total of 274 patients with DM, who were recruited from Chiba University Hospital. Patients with DM were followed up for an observational period of 4.93 years (mean: 4.93 years, SD: 2.77 years, max: 9.58 years, min: 0.07 years). The primary endpoint of this study was to examine whether PCSK9-Abs can be a prognostic marker for overall mortality among the patients with diabetes. The secondary endpoint was to examine the relationship between PCSK9-Abs and clinical parameters.

### Ethics declarations

The present study was approved by the local Ethical Review Board of Chiba University, Graduate School of Medicine (Chiba, Japan) (approval number 973-2018-320), and the International University of Health and Welfare (Chiba, Japan) (approval number 21-Im-037). Serum was collected from participants who had provided written informed consent by following the protocols approved by their institutional ethical committees.

### Collection of serum samples

Samples with total of 3–5 ml of blood were collected using blood collection tubes containing blood coagulation accelerant. Collected blood samples were left for 1 h at room temperature and each serum sample was centrifuged at 3,000*g* for 10 min at 4 °C. The serum samples were then stored at – 80 °C until further use. Repeated thawing and the freezing of the serum samples was avoided.

### Purification of recombinant proteins

Glutathione S-transferases (GST) and GST-fused PCSK9 proteins were purified by affinity chromatography using glutathione–Sepharose columns (GE Healthcare Life Sciences), as previously described^1,5^. And these GST and GST-fused PCSK9 proteins were used for measuring PCSK9 Abs by using an amplified luminescence proximity homogeneous assay-linked immunosorbent assay (AlphaLISA).

### Measurement of serum PCSK9-Abs and -protein levels

The levels of serum PCSK9-Abs and -protein were measured using an AlphaLISA for PCSK9. AlphaLISA was conducted with 384-well microtiter plates (white opaque OptiPlate™, PerkinElmer) containing 2.5 μL of 1/100-diluted sera and 2.5 μL of GST or GST-fusion proteins (10 μg/mL) in AlphaLISA buffer (25 mM HEPES, pH 7.4, 0.1% casein, 0.5% Triton X-100 of 1 mg/mL dextran-500, and 0.05% Proclin-300) according to the manufacturer’s instructions (PerkinElmer, http://www.perkinelmer.com/lab-solutions/resources/docs/GDE_ELISA-to-AlphaLISA.pdf). The reaction mixture was incubated at room temperature for 6–8 h, after which anti-human IgG-conjugated acceptor beads (2.5 μL of 40 μg/mL) and glutathione-conjugated donor beads (2.5 μL of 40 μg/mL) were added, and the mixture was incubated for a further 7–21 days at room temperature in the dark. Chemical emission at 607–623 nm (Alpha photon count), which represents the antigen–antibody binding level, was read on an EnSpire Alpha microplate reader (PerkinElmer). Specific reactions were estimated by subtracting the alpha values of the GST control from the values of the GST-PCSK9 proteins. These methods were precisely described before^1–3^.The PCSK9-Abs titers are measured in alpha photon counts and have no units.

### Statistical analysis

The Mann–Whitney U test was used to determine significant differences between the two groups. There is no guarantee that novel serum markers are distributed normally, therefore we used non-parametric tests. Survival curves were plotted using the Kaplan–Meier method and compared using the log-rank test. The Cox proportional hazards model was used to evaluate significant predictors. Cutoff values were determined to Youden index, which maximizes the sum of sensitivity and specificity. All statistical analyses were performed using Excel software, SPSS (IBM SPSS Statistics) and GraphPad Prism 5 (GraphPad Software, Inc.). All tests were two-tailed, and p-values < 0.05 were considered statistically significant.

### Ethics approval and consent to participate

The present study was conducted in accordance with the ethical principles of the Declaration of Helsinki and approved by our ethical committee. The patients understood the study aims and methods and provided written informed consent.

## Results

### PCSK9-Abs were increased in patients with DM compared to HDs

**Figure 1:**
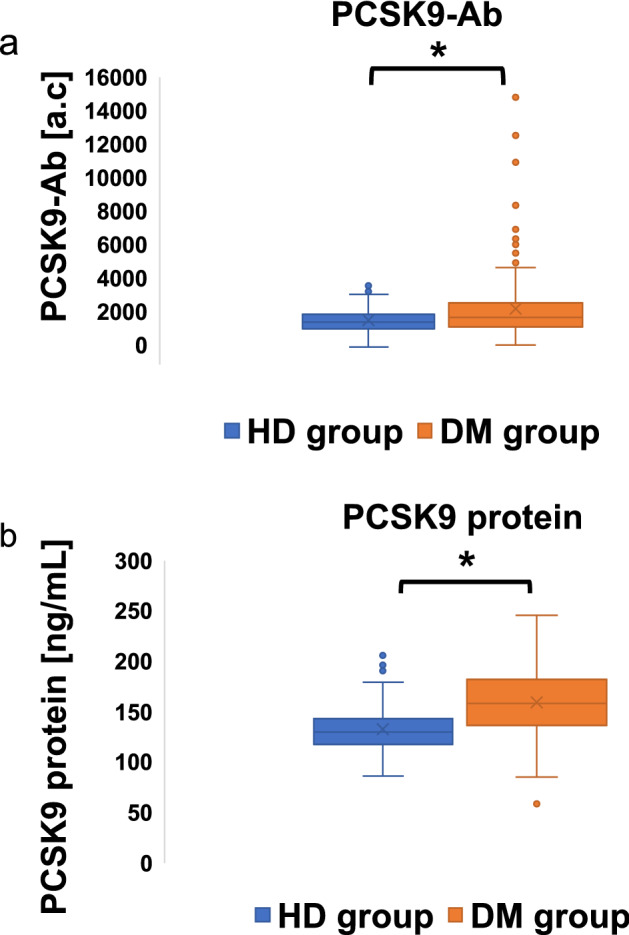
PCSK9-Abs and PCSK protein were increased in patients with DM compared to HDs. Serum levels of proprotein the convertase subtilisin/kexin type 9 antibodies (PCSK9-Abs) (**a**) and PCSK-protein (**b**) examined by amplified luminescence proximity homogeneous assay-linked immunosorbent assay (AlphaLISA) are shown using a box-whisker. The box plots display the 10th, 20th, 50th, 80th, and 90th percentiles. PCSK9-Ab levels in the HD group and DM group were 1452 ± 691 and 2345 ± 3842, respectively (*p < 0.008), and those PCSK9 protein levels were 133 ± 23 ng/ml and 159 ± 33 ng/ml, respectively (*p < 0.001). DM: diabetes mellitus, HD: healthy donor, a.c: Alpha counts.

Table 1 shows the characteristics of the HDs and DM groups included in the analysis. The proportions of patients with type 2 and type 1 diabetes were 89.8% and 10.2%, respectively. Statins were used in 62% of the patients. The samples were obtained before human monoclonal PCSK9-Abs were available on the market. Therefore, patients using human monoclonal PCSK9-Abs were not included in this study. We compared PCSK9-Abs and protein levels between the HDs and DM groups and found that they were significantly higher in the DM group than HDs (PCSK9 Abs, HDs: DM = 1452 ± 691.1: 2345 ± 3842, p < 0.01, PCSK9 protein, HDs: DM = 133 ± 23 ng/ml: 159 ± 33 ng/ml, p < 0.01) respectively as shown in Fig. 1a, b.

**Table 1:** Characteristics of patients included in the analysis.

	HD group (N = 109)		DM group (N = 274)		
	Mean	SD	Mean	SD	p-value
PCSK9-Abs (a.c)	1452	691.1	2345	3482	0.008
PCSK9-protein (ng/mL)	133	23	159	33	< 0.001
Age (year)	58.0	5.8	63.1	12.1	< 0.001
Height (cm)	163.2	9.1	162.6	9.2	0.538
Weight (kg)	63.5	11.9	65.5	14.3	0.184
BMI (kg/m^2^)	23.7	3.2	24.6	4.2	0.034
LDL-C (mg/dL)	128.6	29.8	106.5	29.9	< 0.001
TC (mg/dL)	211.1	30.4	182.9	32.6	< 0.001
TG (mg/dL)	109.5	107.2	153.7	111.2	< 0.001
HDL-C (mg/dL)	67.5	16.8	60.7	17.8	< 0.001
nonHDL-C (mg/dL)	143.6	31.7	123.4	35.0	< 0.001
HbA1c (%)	5.6	0.3	6.7	1.1	< 0.001
TP (g/dL)	7.2	0.4	7.1	0.6	0.348
Alb (g/dL)	4.4	0.3	4.6	3.3	0.502
AST (U/L)	23.4	9.0	26.4	14.0	0.038
ALT (U/L)	23.2	16.3	26.1	17.8	0.136
γ-GTP (U/L)	40	44.1	52.63	87.0	0.148
ALP (U/L)	201.9	58.9	247	94.2	< 0.001
LDH (U/L)	176.1	33.6	206	72.7	< 0.001
UA (mg/dL)	5.4	1.2	5.4	1.4	0.830

A total of 109 healthy donor (HD) blood samples were collected from the Port Square Kashiwado Clinic. A total of 274 patients with diabetes (DM) were recruited from Chiba University Hospital. Serum samples were collected from patients who had provided informed consents. A distribution of 109 HD serum samples is 60 male and 50 female. A distribution of 274 patients with DM serum samples is 155 male and 119 female.

The Mann–Whitney U test was used to determine significant differences between the two groups.

PCSK9-Ab: proprotein convertase subtilisin/kexin type 9 antibody, a.c: Alpha photon counts, BMI: body mass index, LDL-C: low density lipoprotein-cholesterol, TC: total cholesterol, TG: triglyceride, HDL-C: high density lipoprotein-cholesterol, HbA1c: hemoglobin A1c, TP: total protein, Alb: albumin AST: aspartate aminotransferase, ALT: alanine aminotransferase, γ-GTP: γ-glutamyl transpeptidase, ALP: alkaline phosphatase, LDH: lactate dehydrogenase, UA: uric acid.

No significant differences in either PCSK9-Abs or PCSK9 protein levels were observed between patients with type 1 or type 2 DM (data not shown).

### Relationship between serum PCSK9 protein and serum PCSK9-Ab concentration

**Figure 2:**
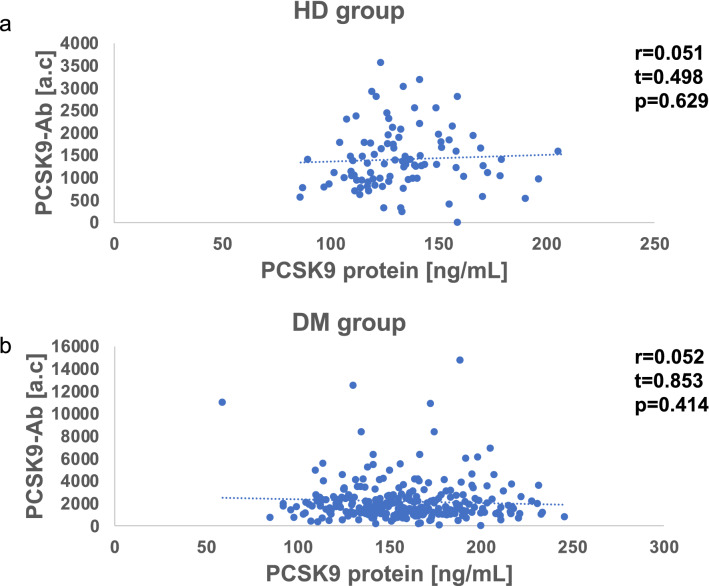
No correlation between the PCSK9 protein and antibody levels were observed neither HDs nor DM. Serum concentration of the convertase subtilisin/kexin type 9 antibodies (PCSK9-Abs) and PCSK-protein were measured by amplified luminescence proximity homogeneous assay-linked immunosorbent assay (AlphaLISA). Scatter dot plots of PCSK9-Abs versus PCSK9 protein in HD group (**a**) and DM group (**b**) are shown. Correlation coefficient (r), test statistic (t), and p-value (p) were calculated by Spearman's correlation analysis. HD: Healthy donors (**a**), DM: patients with diabetes (**b**), a.c: Alpha counts.

We next investigated the association between blood PCSK9 protein levels and blood PCSK9-Ab levels. No correlation was found between the two parameters (HDs: r = 0.05, p = 0.63, DM: r = 0.05, p = 0.41) (Fig. 2a, b).

Even after combining the HD and DM groups, there was no significant relationship between PCSK9-Abs and PCSK9 protein (r = 0.09, p = 0.10).

### Relationship between serum PCSK9 protein, PCSK9-Abs, and clinical laboratory tests

We found no correlation between blood PCSK9-Abs, PCSK9 protein and serum lipid levels (Supplemental Tables 1 and 2). PCSK9-Abs negatively correlated with total protein (r = − 0.134, p = 0.03) and platelet counts (r = − 0.127, p = 0.075), with the latter being non-significant. While PCSK9 protein levels correlated positively with cholinesterase (r = 0.242, p = 0.011), they showed a negative correlation with blood glucose (r = − 0.153, p = 0.03). The level of cholinesterase indicates the nutritional status, and high levels of cholinesterase are reportedly related to hyperlipidemia^24^. Therefore, overnutrition may lead to higher PCSK9 protein levels, which might lead to increased LDL-C. Furthermore, PCSK9 KO mice show increased blood glucose^17^; therefore, the PCSK9 protein might negatively influence blood glucose.

### Relationship between blood PCSK9-Abs and survival prognosis

Since PCSK9-Ab levels were higher in the DM group than in the HD group, we focused on patients with DM. After measuring PCSK9-Abs in the blood of patients with diabetes, they were followed up for an observational period of 4.93 years (mean: 4.93 years, SD: 2.77 years, max: 9.58 years, min: 0.07 years). As a result, 99.6% of the cases were followed up, of which 33 deaths were confirmed. PCSK9-Abs were not associated with the onset of myocardial infarction (MI), stroke onset, or cancer in patients with DM (Table 2).

**Table 2:** Higher PCSK9 antibodies were associated with death in patient with diabetes.

	n	Mean	SD ±	Positive	Two-sided
				rate (%)	Probability
Alive	238	2052.72	1632.44	12.09	1.34E-13
Death	33	4472.82	8805.60		
Non-MI, CI	259	2319.52	3551.63	4.40	0.425
MI + CI	12	3142.83	1436.12		
Non-Cancer	245	2387.60	3653.00	9.52	0.561
Cancer	26	1968.77	946.23		

We had serum data for all 274 patients at the beginning of the study. However, we lacked PCSK9-Ab data for two patients at follow-up. One patient was unable to determine whether he was alive or dead. Finally, we had data for 271 patients at follow-up, which corresponded to 98.9%.

MI: myocardial infarction, CI: cerebral infarction, Any events: one event of death, MI, or CI having occurred. In each event category, All tests were two-tailed, and P-values < 0.05 were considered statistically significant.

Regarding the causes of death, 2 patients out of 7 with MI, 1 patient out of 6 with cerebral infarctions (CI), and 11 patients out of 26 with cancer died. One patient who died with lung cancer had both MI and CI. The causes for the remaining fatalities (n = 18) were unknown.

We found no significant relationship between death and PCSK9 protein levels; however, higher PCSK9-Ab levels were closely associated with the increased mortality (Table 2).

**Figure 3:**
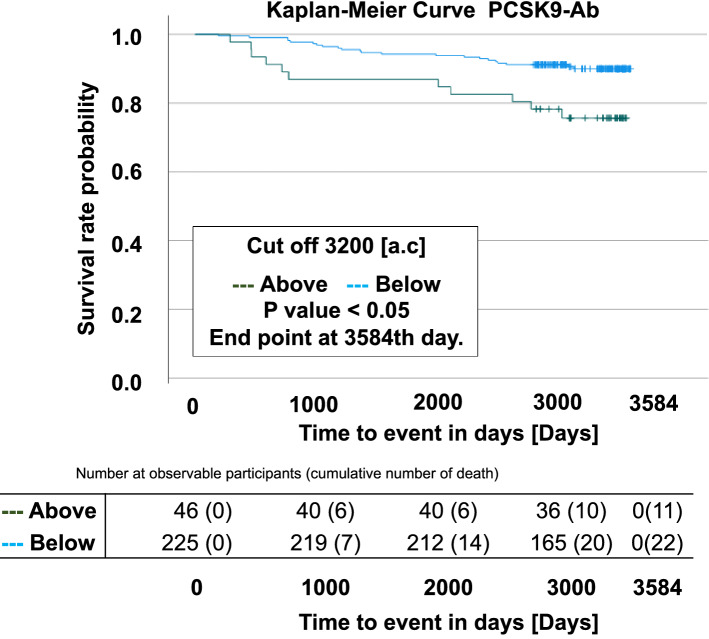
Relationship between blood PCSK9-Abs and survival prognosis. Among the patients with diabetes, 33 deaths were confirmed during observational period (mean: 4.93 years, SD: 2.77 years, Max: 9.58 years, Min: 0.07 years). Since the convertase subtilisin/kexin type 9 antibodies (PCSK9-Abs) levels were higher in the dead patients than alive. The patients were divided into two groups according to the cut-off, calculated as 3200 using Youden’s index, and mortality rates were compared using Kaplan–Meier method and compared using the log-rank test. Blue line indicates PCSK9-Ab level < 3200. Green line indicates PCSK9-Ab level ≥ 3200.

In addition, the patients were divided into two groups according to the cut-off value which was calculated as 3200 using Youden’s index, and mortality rates were compared using Kaplan–Meier curves. The results showed a significantly higher mortality rate in the group with a PCSK9-Ab level > 3200 (log rank test: p = 0.005) (Fig. 3).

Next, we investigated the existence of confounders in the relationship between PCSK9-Ab levels and mortality. We performed Cox regression analysis with mortality as the dependent variable and PCSK9-Ab, LDL-C, age, statin use (with or without), and sex as covariates. We found significant associations between mortality and PCSK9-Ab levels (below 3200, hazard ratio [HR]: 0.404, 95% confidence interval [CI] 0.183–0.891, p = 0.025), sex (HR: 2.305, 95% CI 1.020–5.209, p = 0.045), and age (HR: 1.051, 95% CI 1.014–1.089, p = 0.006) (Table 3). Then, we performed Cox regression analysis with PCSK9-Abs as the dependent variable and LDL-C, age, statin and sex as covariates. We found significant associations only between PCSK9-Abs and age (HR: 1.047, 95% CI 1.009–1.087, p = 0.016) (Table 4).

**Table 3:** Multivariate analysis for mortality-related factors.

	n	%	p-value	HR	95%Cl
PCSK9-Ab (Below 3200)	225	82.1	0.025	0.404	0.183–0.891
Without Statin	167	39.1	0.069	2.024	0.945–4.333
Sex	153 (male)	56.3 (male)	0.045	2.305	1.020–5.209
Age	272	–	0.006	1.051	1.014–1.089
LDL-C	258	–	0.635	1.003	0.990–1.016

We performed Cox regression analysis with mortality as the dependent variable and PCSK9-Ab, LDL-C, age, statin **(with or without)**, and sex as the covariates.

LDL-C; low-density lipoprotein cholesterol, HR: hazard ratio, CI: confidence interval.

**Table 4:** Multivariate analysis for PCSK9-Ab-related factors.

	n	%	p-value	HR	95%Cl
LDL-C	258	–	0.805	0.998	0.985–1.012
Age	273	–	0.016	1.047	1.009–1.087
Statin	167	61.4	0.339	1.446	0.679–3.076
Sex	153 (male)	56.3 (male)	0.051	2.273	0.997–5.178

We performed Cox regression analysis with PCSK9-Ab as the dependent variable and LDL-C, age, statin, and sex as the covariates.

LDL-C; low-density lipoprotein cholesterol, HR: hazard ratio, CI: confidence interval.

Statin use tended to be associated with mortality (relative risk: 0.45), without statistical significance. However, PCSK9-Ab levels did not differ with or without statin use (with statin: 2134 ± 1798; without statin: 2639 ± 4922; p = 0.23). Age was also not directly associated with PCSK9-Ab levels (Supplemental Table 1). Clinical parameters of blood tests other than PCSK9 Ab levels were not found to be associated with mortality in this analysis (Table 4). We therefore suggest that PCSK9-Ab levels may be used as a novel marker for the prognosis of patients with diabetes.

## Discussion

Our results show that PCSK9-Abs are higher in patients with DM than in HDs. To the best of our knowledge, our study is the first to demonstrate that PCSK9-Abs are significantly associated with prognosis for overall survival in patients with diabetes. These results indicated that PCSK9-Abs can be used as prognostic markers for overall survival in patients with diabetes, independent from age and statin use.

The PCSK9 protein is produced intracellularly, and mature PCSK9 is released into the extracellular space. PCSK9 binds with LDL receptors, which are transported to lysosomes and degraded via amyloid precursor-like 2 proteins^25^.

Gain-of-function mutations in the *PCSK9* gene result in increased blood PCSK9 protein levels, LDL receptor degradation, and increased blood LDL-C, which causes familial hypercholesterolemia. Regardless of gene mutations, increased levels of blood PCSK9 protein led to increased levels of LDL-C and may be involved in the development of arteriosclerotic disease. Cross-sectional studies examining the association between blood PCSK9 protein levels and arteriosclerotic disease have reported conflicting results^11–14^. It is difficult to predict the development of arteriosclerotic diseases and subsequent cardiovascular events by measuring PCSK9 protein concentration at a single timepoint because PCSK9 protein concentration is influenced by various factors such as concurrent drug administration^26,27^ and nutritional status^16^. Therefore, antibody markers are expected to serve as memory markers that reflect past situations. In fact, our study did not show any association between PCSK9 protein and PCSK9-Ab levels measured at the same point in time. PCSK9 Abs can be produced even if the PCSK9 protein blood levels are low, and its production increases dramatically with repeated exposure to the PCSK9 protein. Since the IgGs of PCSK9 Abs are more stable in the blood than the PCSK9 protein itself, it is not necessary for the levels of PCSK9-Abs to be related with PCSK9 protein levels. This also indicates that PCSK9-Ab level is not merely an indicator of the amount of PCSK9 protein present at a given time point. We found a weak negative association between blood PCSK9-Abs and platelet count (Supplemental Table 1). Recently, it was reported that platelet derived PCSK9 may increase platelet function and play a role in the development of arterial stiffness, and neutralizing antibodies to PCSK9 may reduce platelet aggregation and inhibit cardiovascular events^28^. Since this study did not examine the association between PCSK9-Abs and platelet function, further studies are needed to understand its biological implications, rather than that between PCSK9 and platelet count.

Recently, it has been reported that PCSK9 is related not only with lipid metabolism but also DM. For instance, some clinical studies reported increased levels of serum PCSK9 protein levels among patients with type 2 DM^18–20^, however there are conflicting data^29^. Indeed, our results also indicated that the PCSK9 protein was associated with DM.

Regarding the PCSK9 function within DM, it has been reported that PCSK9 KO mice have impaired insulin secretion and increased blood glucose^17^. The lack of PCSK9 in mice increased expression of LDL-R within the islets of Langerhans, which then led to increased LDL-C levels and impaired insulin secretion^17^. Furthermore, PCSK9 and LDL-R double KO mice did not show impaired insulin secretion, although they show low levels of LDL-C^17^. Nevertheless, these results indicated that PCSK9 protein is involved in glucose metabolism. Therefore, we focused on the relationship between PCSK9-Abs, PCSK9 protein, and DM.

In this study, we investigated the relationship between PCSK9-Ab titers and survival prognosis. Initially, we expected that if a PCSK9-Abs acted as a neutralizing antibody, the LDL-C level and cardiovascular events would decrease in the group with a high PCSK9-Ab titer. However, the number of cardiovascular events was not increased in the group with high PCSK9-Ab titers. We also analyzed the association between PCSK9-Abs and serum lipid levels with or without statins, but found no association. At present, the reason for the higher mortality rate in the group with high PCSK9-Ab titers remains unclear; however, this could be related to past dyslipidemia and platelet function. An association between cancer and PCSK9-Abs has been reported^5^, therefore, an undiscovered cancer with high PCSK9-Ab level may be related to the poor prognosis among the patients with DM.

Although, PCSK9-Abs were not related with serum lipid levels, it is very intriguing that PCSK9-Abs were increased in the patient with diabetes. PCSK9 KO mice increased blood glucose due to impaired insulin secretion but not increased insulin resistance^17^ and gain of function gene mutation in human increase the risk of development type 2 diabetes^30^. These results clearly indicated that neutralizing PCSK9 function related with diabetes. Therefore, PCSK9-Abs measured in this study might have more affinity to the PCSK9 secreted from islet of β cells than the PCSK9 secreted from the liver. These speculations need to be investigated in the future.

This study has some limitations. First, we included only patients with diabetes for further analysis, and whether PCSK9-Abs and survival prognosis are related in other groups, such as HDs, is unclear. Second, we performed only one measurement; it is necessary to examine the changes over time in similar cases. Third, although we could speculate on the neutralizing function of the PCSK9-Abs, it remains unclear. Fourth, although it has been reported that the concentration of circulating PCSK9 might be a predictor for the development of type 2 DM^31^, we could not follow up the HD population; consequently, we do not know whether their PCSK9-Ab levels will relate with the new onset of diabetes in this population.

## Conclusions

In conclusion, this study suggests that PCSK9-Abs may be a novel marker for DM, while being useful as a predictive marker for overall survival of patients with DM. Its usefulness as a prognostic marker should be verified in the future with large patient numbers.

## Supplementary Information

Supplementary Information.

## Data Availability

The datasets used and/or analysed during the current study available from the corresponding author on reasonable request.

## Abbreviations

PCSK9: Proprotein convertase subtilisin/kexin type 9
AlphaLISA: Amplified luminescence proximity homogeneous assay-linked immunosorbent assay
a.c: Alpha counts
BMI: Body mass index
CI: Cerebral infarction
DM: Diabetes mellitus
HDs: Healthy donors
LDL: Low-density lipoprotein
HDL: High density lipoprotein
HbA1c: Hemoglobin A1c
MI: Myocardial infarction
TC: Total cholesterol
TG: Triglyceride
Alb: Albumin
AST: Aspartate aminotransferase
ALT: Alanine aminotransferase
γ-GTP: γ-Glutamyl transpeptidase
ALP: Alkaline phosphatase
LDH: Lactate dehydrogenase
UA: Uric acid

## References

[1] Hiwasa T (2021). Serum anti-DIDO1, anti-CPSF2, and anti-FOXJ2 antibodies as predictive risk markers for acute ischemic stroke. BMC. Med..

[2] Li SY (2021). Serum anti-AP3D1 antibodies are risk factors for acute ischemic stroke related with atherosclerosis. Sci. Rep..

[3] Yoshida Y (2020). Elevated levels of autoantibodies against DNAJC2 in sera of patients with atherosclerotic diseases. Heliyon.

[4] Nanami T (2021). Prevalence of serum galectin-1 autoantibodies in seven types of cancer: A potential biomarker. Mol. Clin. Oncol..

[5] Ito M (2021). Association of serum anti-PCSK9 antibody levels with favorable postoperative prognosis in esophageal cancer. Front. Oncol..

[6] Abifadel M (2003). Mutations in PCSK9 cause autosomal dominant hypercholesterolemia. Nat. Genet..

[7] Maxwell KN, Soccio RE, Duncan EM, Sehayek E, Breslow JL (2003). Novel putative SREBP and LXR target genes identified by microarray analysis in liver of cholesterol-fed mice. J. Lipid. Res..

[8] Maxwell KN, Breslow JL (2004). Adenoviral-mediated expression of Pcsk9 in mice results in a low-density lipoprotein receptor knockout phenotype. Proc. Natl. Acad. Sci. USA.

[9] Cohen J (2005). Low LDL cholesterol in individuals of African descent resulting from frequent nonsense mutations in PCSK9. Nat. Genet..

[10] Cohen JC, Boerwinkle E, Mosley TH, Hobbs HH (2006). Sequence variations in PCSK9, low LDL, and protection against coronary heart disease. N. Engl. J. Med..

[11] Werner C, Hoffmann MM, Winkler K, Bohm M, Laufs U (2014). Risk prediction with proprotein convertase subtilisin/kexin type 9 (PCSK9) in patients with stable coronary disease on statin treatment. Vascul. Pharmacol..

[12] Gencer B (2016). Prognostic value of PCSK9 levels in patients with acute coronary syndromes. Eur. Heart. J..

[13] Zhou Y, Chen W, Lu M, Wang Y (2021). Association between circulating proprotein convertase subtilisin/kexin type 9 and major adverse cardiovascular events, stroke, and all-cause mortality: systemic review and meta-analysis. Front. Cardiovasc. Med..

[14] Vlachopoulos C (2016). Prediction of cardiovascular events with levels of proprotein convertase subtilisin/kexin type 9: A systematic review and meta-analysis. Atherosclerosis.

[15] Arsenault BJ (2014). PCSK9 levels in abdominally obese men: association with cardiometabolic risk profile and effects of a one-year lifestyle modification program. Atherosclerosis.

[16] Costet P (2006). Hepatic PCSK9 expression is regulated by nutritional status via insulin and sterol regulatory element-binding protein 1c. J. Biol. Chem..

[17] Da Dalt L (2019). PCSK9 deficiency reduces insulin secretion and promotes glucose intolerance: The role of the low-density lipoprotein receptor. Eur. Heart. J..

[18] Lakoski SG, Lagace TA, Cohen JC, Horton JD, Hobbs HH (2009). Genetic and metabolic determinants of plasma PCSK9 levels. J. Clin. Endocrinol. Metab..

[19] Nekaies Y, Baudin B, Kelbousi S, Sakly M, Attia N (2015). Plasma proprotein convertase subtilisin/kexin type 9 is associated with Lp(a) in type 2 diabetic patients. J. Diabetes. Complications..

[20] Ibarretxe D (2016). Circulating PCSK9 in patients with type 2 diabetes and related metabolic disorders. Clin. Investig. Arterioscler..

[21] Koye DN, Magliano DJ, Nelson RG, Pavkov ME (2018). The global epidemiology of diabetes and kidney disease. Adv. Chronic. Kidney. Dis..

[22] Beckman JA, Creager MA, Libby P (2002). Diabetes and atherosclerosis: Epidemiology, pathophysiology, and management. JAMA.

[23] Berezin A (2016). Metabolic memory phenomenon in diabetes mellitus: Achieving and perspectives. Diabetes. Metab. Syndr..

[24] Oda E (2015). Associations between serum cholinesterase and incident hyper-LDL cholesterolemia, hypertriglyceridemia and hypo-HDL cholesterolemia as well as changes in lipid levels in a health screening population. Atherosclerosis.

[25] DeVay RM, Shelton DL, Liang H (2013). Characterization of proprotein convertase subtilisin/kexin type 9 (PCSK9) trafficking reveals a novel lysosomal targeting mechanism via amyloid precursor-like protein 2 (APLP2). J. Biol. Chem..

[26] Sahebkar A, Simental-Mendia LE, Guerrero-Romero F, Golledge J, Watts GF (2015). Effect of statin therapy on plasma proprotein convertase subtilisin kexin 9 (PCSK9) concentrations: A systematic review and meta-analysis of clinical trials. Diabetes. Obes. Metab..

[27] Sahebkar A (2014). Circulating levels of proprotein convertase subtilisin kexin type 9 are elevated by fibrate therapy: A systematic review and meta-analysis of clinical trials. Cardiol. Rev..

[28] Petersen-Uribe A (2021). Platelet-derived PCSK9 is associated with LDL metabolism and modulates atherothrombotic mechanisms in coronary artery disease. Int. J. Mol. Sci..

[29] Brouwers MC (2011). Plasma proprotein convertase subtilisin kexin type 9 is not altered in subjects with impaired glucose metabolism and type 2 diabetes mellitus, but its relationship with non-HDL cholesterol and apolipoprotein B may be modified by type 2 diabetes mellitus: The CODAM study. Atherosclerosis.

[30] Saavedra YGL, Dufour R, Baass A (2015). Familial hypercholesterolemia: PCSK9 InsLEU genetic variant and prediabetes/diabetes risk. J. Clin. Lipidol..

[31] Peng J, Zhu CG, Li JJ (2021). The predictive utility of circulating PCSK9 levels on diabetes mellitus. Cardiovasc. Diabetol..

