# Supplementary material for: Association of high proprotein convertase subtilisin/kexin type 9 antibody level with poor prognosis in patients with diabetes: a prospective study
Source: Sci Rep. 2023 Apr 3;13:5391. doi: 10.1038/s41598-023-32644-y (PMC10070486; doi:10.1038/s41598-023-32644-y)
Supplement: Supplementary file 1 — Supplementary Information. [file 41598_2023_32644_MOESM1_ESM.pdf]

	PCSK9-Ab (N=274)			
	t	p	95%CI	
Age [year]	-0.43	0.67	-74.7	48.3
Height [cm]	-1.96	0.06	-543.5	6.9
Weight [kg]	1.90	0.06	-17.4	669.3
BMI [kg/m^2]	-1.80	0.08	-1736.5	93.9
LDL-C [mg/dL]	0.21	0.84	-32.0	39.5
TC [mg/dL]	0.19	0.85	-37.1	45.0
TG [mg/dL]	-0.30	0.77	-5.5	4.1
HDL-C [mg/dL]	-0.26	0.80	-48.8	37.6
HbA1c [%]	-0.20	0.84	-465.4	381.2
TP [g/dL]	-0.10	0.92	-1073.5	973.8
Alb [g/dL]	-1.67	0.10	-3361.1	310.4
AST [U/L]	0.10	0.32	-41.0	120.5
ALT [U/L]	-1.10	0.28	-97.0	28.4
γ-GTP [U/L]	-0.19	0.85	-6.7	5.5
ALP [U/L]	-0.38	0.70	-6.3	4.3
LDH [U/L]	-0.37	0.71	-5.1	3.5
UA [mg/dL]	-0.42	0.70	-476.5	312.0

Supplemental Table 1. Correlation between blood PCSK9-Ab and clinical parameters in patient with diabetes.

Correlations were calculated using Spearman's correlation and logistic regression analysis. All statistical analyses were performed using SPSS software. All tests were two-tailed, and P-values <0.05 were considered statistically significant.

	PCSK9-Ag (N=274)			
	t	p	95%CI	
Age [year]	0.87	0.39	-1192.2	3007.2
Height [cm]	-0.06	0.95	-9714.3	9147.7
Weight [kg]	-0.22	0.82	-13068.8	10439.5
BMI [kg/m^2]	0.25	0.80	-27358.7	35292.7
LDL-C [mg/dL]	-1.36	0.18	-2026.3	386.2
TC [mg/dL]	1.30	0.20	-491.0	2308.3
TG [mg/dL]	-0.56	0.58	-208.8	117.7
HDL-C [mg/dL]	-1.59	0.12	-2648.1	309.7
HbA1c [%]	0.22	0.83	-12902.6	16076.7
TP [g/dL]	0.18	0.86	-31904.8	38143.7
Alb [g/dL]	0.25	0.80	-54521.1	70293.3
AST [U/L]	0.63	0.53	-1901.0	3634.2
ALT [U/L]	-1.14	0.26	-3368.9	922.5
γ-GTP [U/L]	0.47	0.64	-160.4	258.3
ALP [U/L]	-0.36	0.72	-212.0	147.0
LDH [U/L]	0.31	0.75	-124.3	170.7
UA [mg/dL]	-0.85	0.40	-19249.3	7766.9

Supplemental Table 2. Correlations between blood PCSK9 protein and clinical parameters in patients with diabetes.

Correlations were calculated using Spearman’s correlation and logistic regression analysis. All statistical analyses were performed using Excel software. All tests were two-tailed, and P-values <0.05 were considered statistically significant.
